# Binding affinity-based intracellular drug detection enabled by a unimolecular cucurbit[7]uril-dye conjugate†

**DOI:** 10.1039/d3cb00131h

**Published:** 2023-09-07

**Authors:** Yanxi Liu, Changming Hu, Julian A. Serna, Frank Biedermann, Pavel A. Levkin

**Affiliations:** a Karlsruhe Institute of Technology (KIT), Institute of Biological and Chemical Systems – Functional Molecular Systems (IBCS-FMS), Hermann-von-Helmholtz-Platz 1 Eggenstein-Leopoldshafen 76344 Germany; b Institute of Pathology and Southwest Cancer Center, Southwest Hospital, Third Military Medical University (Army Medical University) and Key Laboratory of Tumor Immunopathology, Ministry of Education of China Chongqing 400038 China; c Karlsruhe Institute of Technology (KIT), Institute of Nanotechnology (INT), Hermann-von-Helmholtz Platz 1 Eggenstein-Leopoldshafen 76344 Germany frank.biedermann@kit.edu; d Karlsruhe Institute of Technology (KIT), Institute of Organic Chemistry (IOC) Kaiserstraße 12 Karlsruhe 76131 Germany levkin@kit.edu

## Abstract

Label-free fluorescence-based chemosensing has been increasingly brought into focus due to its simplicity and high sensitivity for intracellular monitoring of molecules. Currently used methods, such as conventional indicator displacement assays (IDAs), pose limitations related to dissociation upon dilution, random diffusion of the released indicators, and high sensitivity to interference by agents from the ambient cellular environment (*e.g.*, salts, enzymes, and proteins). Herein we report a potentially widely applicable strategy to overcome the limitations of conventional IDAs by employing a macrocyclic cucurbit[7]uril (CB7) host covalently coupled to a nitrobenzoxadiazole (NBD) fluorescent dye (CB7-NBD conjugate). As a proof of concept, we demonstrated that the CB7-NBD unimolecular conjugate responded to various target analytes even in the complex live cell system. Moreover, the sensing system was compatible with fluorescence imaging, fluorescence-assisted cell sorting (FACS), and fluorescence spectrometry with a microplate reader. These experiments demonstrated an application of covalently bound unimolecular CB7-NBD conjugate as a sensor for detecting diverse analytes in the intracellular compartment of live cells.

## Abstract 2

We demonstrate an application of macrocyclic cucurbit[7]uril (CB7) host covalently coupled to a nitrobenzoxadiazole (NBD) fluorescent dye (CB7-NBD conjugate) as a sensor for detecting diverse analytes in the intracellular compartment of live cells.

## Graphical abstract

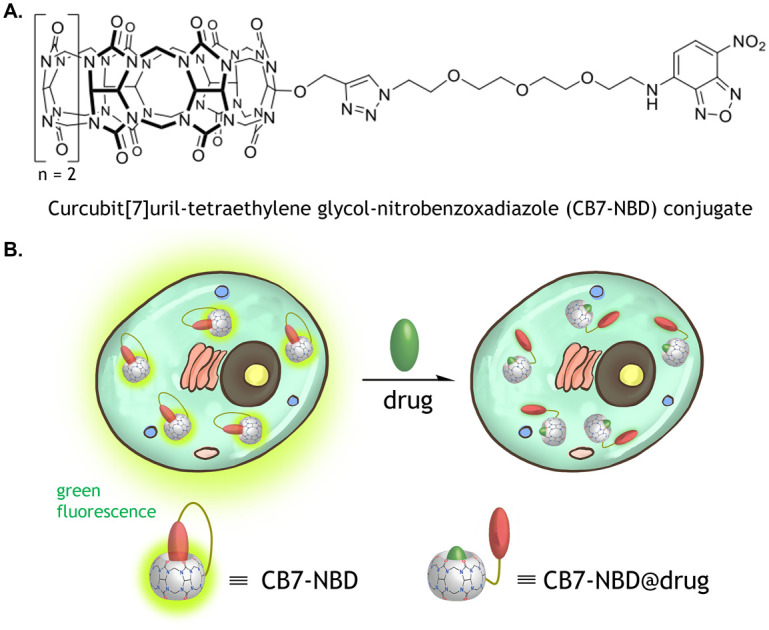

## Introduction

In the clinical setting, the therapeutic success of difficult-to-manage drugs is conditioned to using the right dose for every patient, both in terms of safety and efficacy. The most widely accepted strategy for determining such a right dose is therapeutic drug monitoring (TDM), in which the concentrations of specific drugs at designated time intervals is measured.^1^ However, TDM is often performed by analyzing whole-blood or plasma samples obtained from patients, which might not necessarily relate to the drug's effect. Consequently, intracellular TDM has become increasingly important, particularly for drugs that exert their action inside cells.^2–4^ Several analytical methods have been implemented for determining intracellular drug concentration, including chromatography- and mass spectrometry-based methods (high-performance liquid chromatography (HPLC) and liquid chromatography–mass spectrometry (LC-MS)),^5,6^ immunoassays,^7,8^ and fluorescence-based techniques.^9,10^ Nevertheless, their limitations significantly hamper their adoption and wide applications in the clinical setting. In particular, LC- and MS-based analytical methods have been demonstrated to be effective for quantifying drug concentrations, but they involve complex procedures and equipment, as well as labour-intensive sample preparation.^11^ Immunoassays are based on the antigen–antibody binding reaction, which can be heavily affected by cross reactants inside living cells, thus making them unsuitable for monitoring small molecule drugs.^12^ Furthermore, both methods lack real-time monitoring or sensing capability.^13^ Among the current real-time drug monitoring strategies, fluorescence imaging is considered one of the most powerful techniques owing to its simplicity, high spatial resolution, adaptability to automated analysis, multiple signal output modes, and high sensitivity.^10^ Accordingly, following the emission signal of inherently fluorescent drug molecules is the ideal real-time monitoring mode, but most drugs are neither fluorescent nor light-absorbent. Moreover, conjugating an additional dye tag to a drug molecule will alter the drug's biological and physicochemical properties, thus affecting its trafficking across cell membranes and its therapeutic efficacy.^13^ Hence, label-free fluorescence-based chemosensing strategies, like indicator displacement assays (IDAs), hold great potential for intracellular drug monitoring.^14^

The IDAs are based on the competition between an indicator (dye) and an analyte (guest) for the binding of a receptor (host), in which the competitive displacement of the indicator by the analyte results in the occurrence of a signal change.^15^ Given the advantages of IDAs, such as avoidance of covalent modification of the analyte with an indicator (dye) and ease of optimization, they have been widely used for sensing ions,^16^ small molecules,^17,18^ enzymes,^19^ and bioorganic analytes in living cells,^20^ as well as for determining host–guest binding constants.^21^ Conventional bimolecular IDAs, however, have some disadvantages: (1) to ensure competitive binding conditions, the binding affinity and working concentrations of indicator and analyte need to be tuned;^22^ (2) the non-covalent interaction between host and guest can be strongly influenced by salts present in solution;^23^ (3) the non-covalent complexation is inherently easy to dissociate upon dilution (dilution induced dissociation);^24^ and more importantly, (4) the released indicator from the displacement assay is stochastically distributed due to the random diffusion, and is not spatially located together with target analytes.^10^ Integrating IDAs with other techniques such as Förster resonance energy transfer (FRET) could, in part, compensate limitations of incomplete complexation-induced fluorescence change. However, the problems related to dilution-induced dissociation and random distribution of released indicator molecules are not solved by this approach.

We hypothesize that the use of covalently bound host-dye conjugates,^18,25^ such as our recently reported cucurbit[7]uril (CB7)-based conjugates,^23^ could be a suitable approach to circumvent the limitations of dilution and random distribution of the released indicator.

**Fig. 1:**
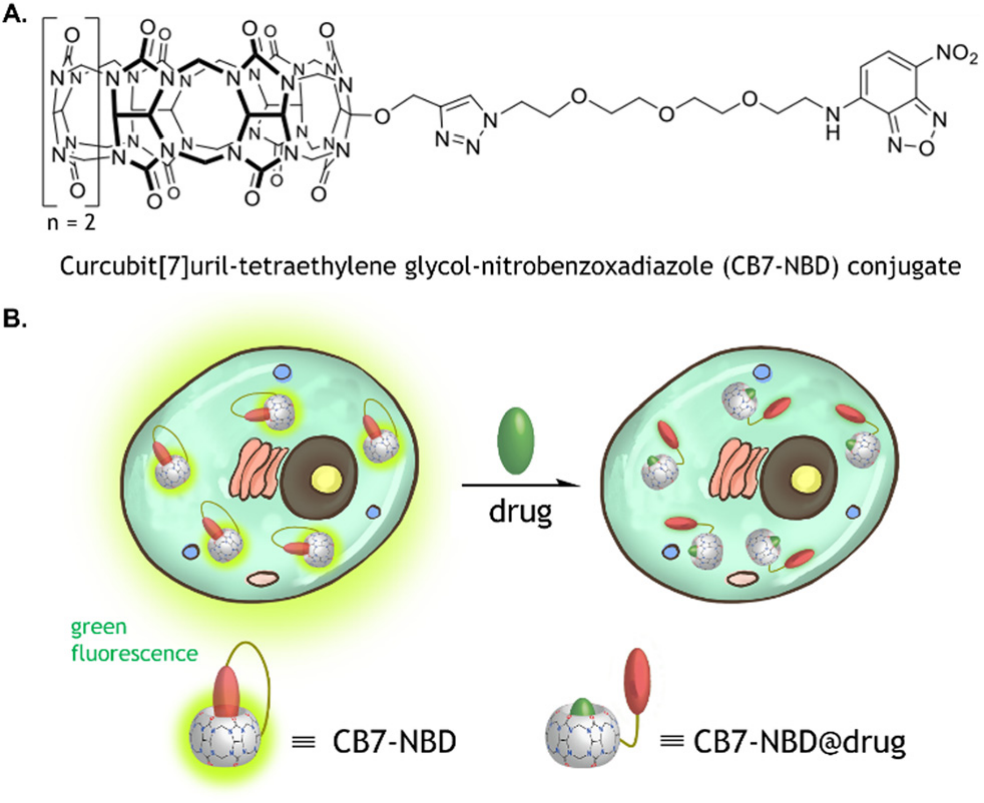
Chemical structure of the CB7-NBD conjugate and schematic illustration of its functionality. (A) Chemical structure of the CB7-NBD conjugate prepared *via* an Azide–Alkyne Huisgen cycloaddition between CB7 and NBD coupled to two terminals of a tetraethylene glycol-azide (NBD-TEG-N3) linker. (B) In its self-encapsulation state, the conjugate emits green fluorescence, which ceases upon displacement of the NBD by a molecule with a higher binding affinity to CB7.

CB7 is a pumpkin-shaped macrocyclic host molecule with two identical carbonyl-lined portals and seven glycoluril units.^26^ It displays relatively strong binding affinity and reasonable selectivity for hydrophobic or positively charged guest molecules.^27^ Additional assets of CB7 comprise its water solubility and the capability of binding to many indicators and biologically relevant analytes. Accordingly, these characteristics have been harnessed for biological applications of CB7, including drug delivery,^28^ chemotherapy,^29^ and bioimaging.^30,31^ We recently reported the cucurbit[7]uril-tetraethylene glycol-nitrobenzoxadiazole (CB7-NBD) conjugate, which displays a strong green fluorescence signal when in a self-association status.^23^ However, in the presence of an analyte with a high affinity for the CB7 cavity, NBD is displaced from the host's cavity, and thus NBD's green fluorescence is diminished (Fig. 1B). Considering the current limitations encountered by conventional IDAs for intracellular drug monitoring, we studied in this work the potential of CB7-based chemosensors for label-free and biocompatible monitoring of small molecules within internal compartments of living systems. In particular, we showed that the CB7-NBD conjugate is biocompatible, can be internalized by human cells, and can be used for tracking the intracellular presence of small molecules by means of fluorescence. Our platform proved compatible with multiple fluorescence-based measuring instruments, such as fluorescence microscopy imaging, fluorescence-activated cell sorting (FACS), and fluorescence spectrometry. These findings open the door for harnessing the biological potential of unimolecular CB7-based chemosensors for label-free sensing of molecules within the intracellular space of living systems.

## Results and discussion

The CB7 was covalently bound to NBD *via* a tetraethylene glycol-azide (TEG-N3) linker to form the unimolecular CB7-NBD conjugate, as previously described (Fig. 1).^23^ The conjugate design took several considerations into account. An azide-terminated NBD-TEG and a propargyl-functionalized monosubstituted CB7 were selected and bound to construct the CB7-NBD conjugate. The NBD dye that possesses a modest affinity for CB7 was selected to track the competition with diverse target analytes. Even though non-covalent CB7 ⊃ dye complexes showed high binding affinities and fast binding kinetics in deionized water,^32^ the non-covalent interaction between host and guest is strongly modulated by competitive cation binding or by dilution, which limits their efficacy for cell-based IDAs.^20^ For instance, we found that the non-covalent CB7 ⊃ acridine orange (AO) and CB7 ⊃ berberine chloride (BC) complexes did not respond to the addition of amantadine to the cell culture medium (Fig. S1 and S2, ESI†). The inability of these non-covalent complexes to detect the compound could be due to their dissociation in the cell culture medium owing to the presence of salts.^23^

**Fig. 2:**
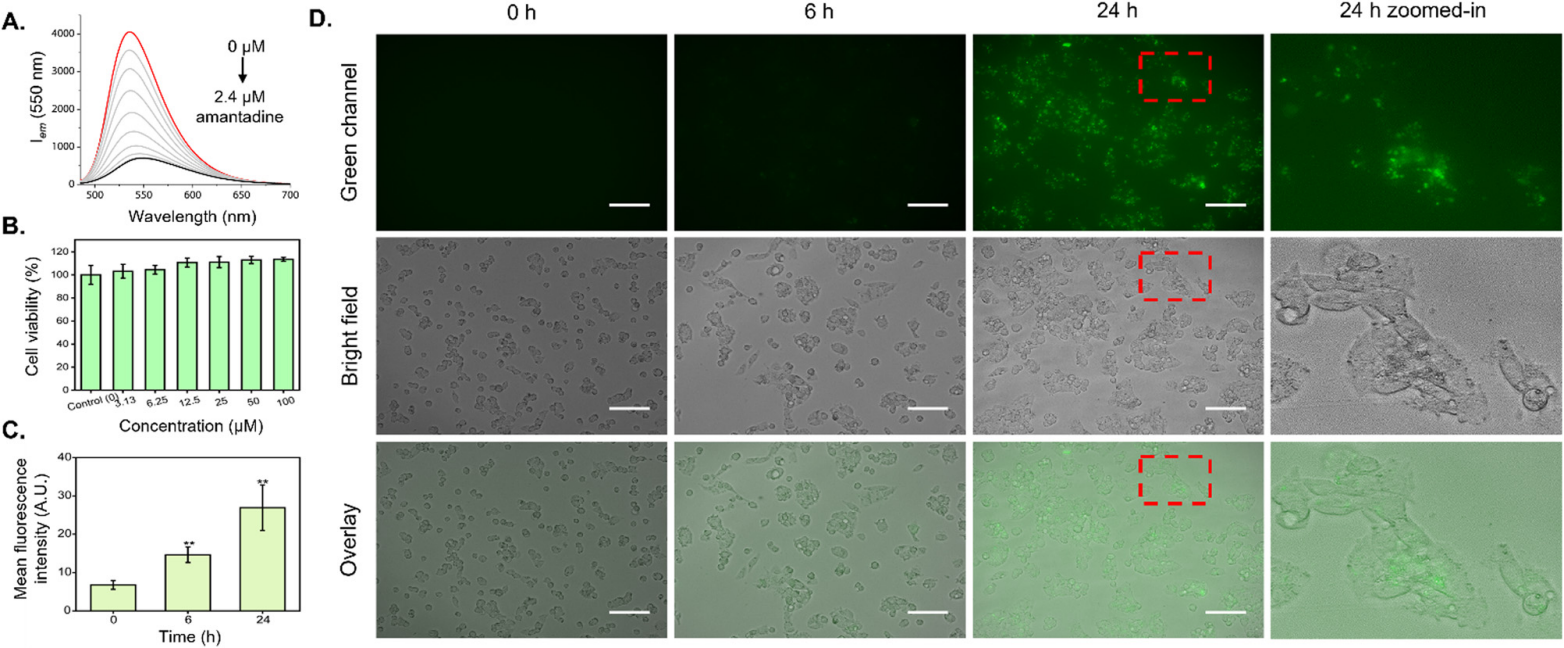
Emission spectra of CB7-NBD in the presence of amantadine, cell viability of HepG2 cells exposed to CB7-NBD, and time-dependent cellular uptake of the CB7-NBD conjugate. (A) The fluorescence emission of the CB7-NBD conjugate (1 μM, *λ*_ex_ = 475 nm) was diminished with exposure to amantadine in a concentration-dependent manner in DMEM cell culture medium. (B) The cell viability of HepG2 cells treated with various concentrations of CB7-NBD (3.13, 6.25, 12.5, 25, 50, and 100 μM) remained comparable to that of the control after 72 h of exposure (*n* = 3), as no statistical difference was observed. (C and D) The HepG2 cells were incubated with CB7-NBD (green) at a concentration of 50 μM for 0 h, 6 h, and 24 h. The cellular uptake was visualized by a fluorescence microscope (magnification 20×, *λ*_ex_ = 470/40 nm, *λ*_em_ = 525/50 nm). Scale bar: 100 μm. The mean fluorescence intensity of CB7-NBD was analyzed by ImageJ software (*n* = 3). ** Indicates *p* < 0.01 when compared with the 0 h group, which was considered statistically significant.

Conversely, the detection of amantadine was feasible (Fig. S3, ESI†) when CB7 and BC were covalently bonded to form the unimolecular CB7-BC conjugate (Fig. 2A). Indeed, the unimolecular CB7-NBD conjugate exhibited an even stronger fluorescence signal change and detection ability of amantadine compared to CB7-BC. Having demonstrated the superiority of the covalently bound CB7-NBD conjugate in cell culture medium, we proceeded to study its cytocompatibility and cellular uptake.

In order to evaluate the compatibility of the CB7-NBD conjugate with live cell biological applications, viability and uptake assays were performed using the established human liver carcinoma cell line HepG2. Firstly, the MTT assay was conducted to study cellular metabolic activity as a direct indicator of cell viability. As shown in Fig. 2B, all evaluated concentrations of the CB7-NBD conjugate (3.13, 6.25, 12.5, 25, 50, and 100 μM) were well tolerated by HepG2 cells after 72 h of exposure, as cell viability remained comparable to that of the control with sole cell culture medium. Secondly, the cellular uptake of CB7-NBD was assessed by signal intensity analysis based on fluorescence microscopy images. Compared to the untreated control, a significant increase in green fluorescence intensity could be observed after 6 and 24 h of incubation (Fig. 2D), which suggested the cellular uptake of CB7-NBD conjugate by HepG2 cells. Moreover, a stronger green fluorescence intensity observed after 24 h exposure compared to the 6 h group suggested a time-dependent conjugate uptake.

**Fig. 3:**
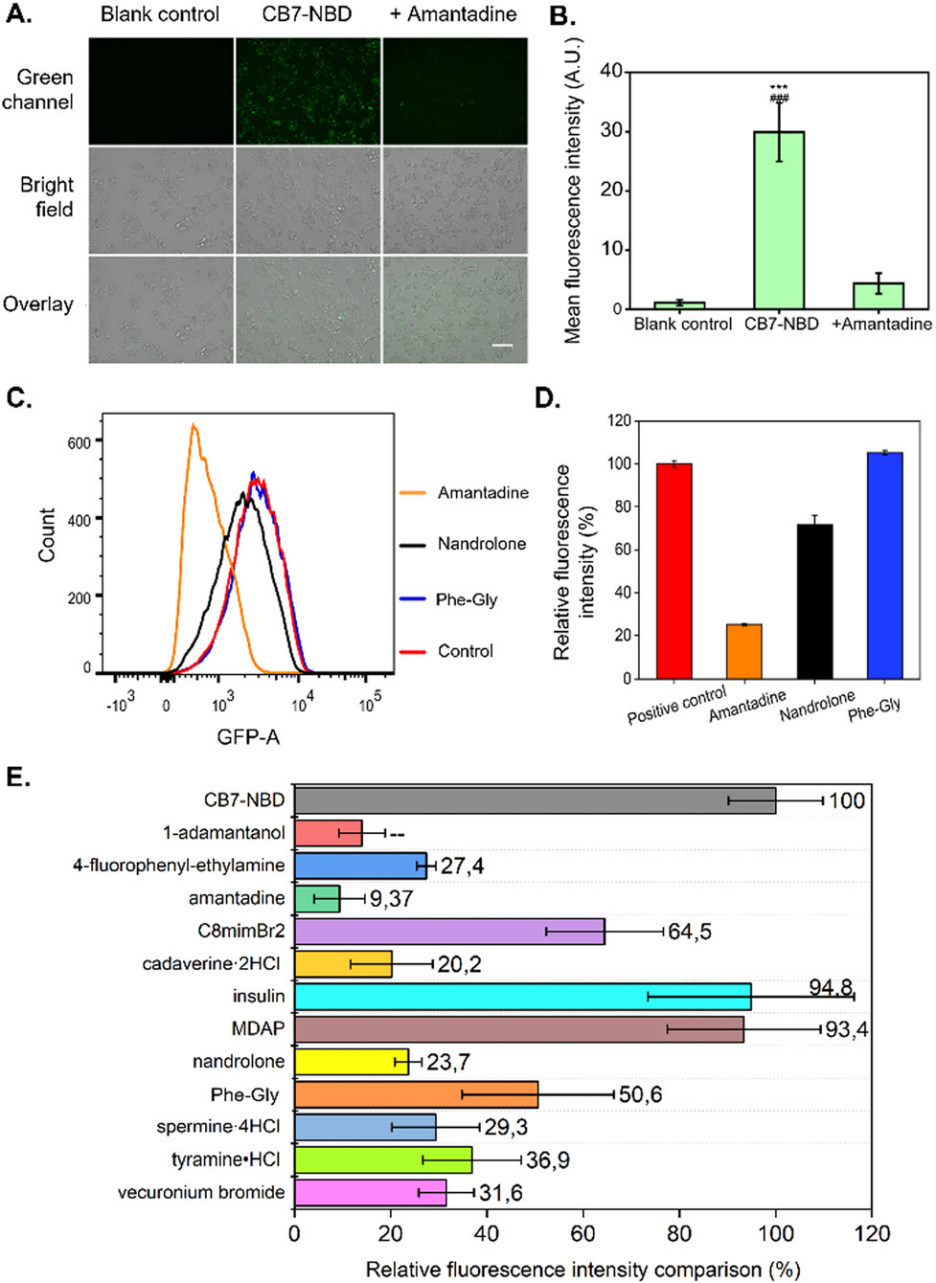
IDAs of CB7-NBD in HepG2 cells obtained by fluorescence imaging, FACS, and fluorescent spectroscopy. (A) The HepG2 cells were incubated with CB7-NBD (green) at a concentration of 50 μM for 24 h before adding 500 μM amantadine and incubated for 15 min. The displacement process was visualized by a fluorescence microscope Keyence at the magnification of 20×. GFP was the fluorescence filter tube used for observation (*λ*_ex_ = 470/40 nm, *λ*_em_ = 525/50 nm). Scale bar: 100 μm. (B) The mean fluorescence intensity of blank control and CB7-NBD before and after adding amantadine were analyzed by ImageJ software (*n* = 3). *** Indicates *p* < 0.001 compared with the blank control group. ### Indicates *p* < 0.001 compared with the amantadine group. (C) FACS analysis results show the fluorescence intensity changes of HepG2 cells incubated with CB7-NBD after exposure to amantadine, nandrolone, and Phe-Gly (Fig. S14–S17, ESI†). (D) The relative fluorescence intensity calculated according to FACS analysis (*n* = 3 for each data set). (E) Fluorescence intensity of HepG2 cells treated with various analytes and their intensity percentage compared to cells treated with CB7-NBD. Cells were first exposed to 50 μM CB7-NBD for 24 h. Then the cells were washed with PBS three times, followed by incubation with 500 μM analyte solution for another 15 min and signal measurement with a fluorescence microplate reader (*n* = 3).

Once the uptake of the CB7-NBD conjugate by HepG2 cells was confirmed *via* fluorescence imaging, we performed a cell-based IDA using amantadine as a small molecule analyte. For this, amantadine was selected as a model molecule because it binds to CB7 with a relatively high log *K*_a_ value (>8 in DMEM media) (Fig. S3, ESI†). Fluorescence microscopy images showed that the fluorescence of the CB7-NBD conjugate was rapidly quenched upon adding amantadine at a 500 μM final concentration (Fig. 3A). This observation can be explained by the typical characteristics of the CB7-NBD sensing system. Firstly, NBD was encapsulated in the cavity of CB7 with a modest binding affinity in the cell culture medium. Secondly, amantadine competitively displaces NBD from the cavity of CB7, thereby quenching the fluorescence of NBD. The mean fluorescence intensity was then quantified from microscopy images to analyze the change in fluorescence signal. Significant differences could be observed between the CB7-NBD group *versus* the blank control and amantadine group (*p* < 0.001), suggesting the successful indicator displacement phenomenon between amantadine and CB7-NBD (Fig. 3B). It is worth noting that we performed time-dependent experiments to evaluate the displacement of the dye in living cells (data not shown), from which an incubation time of 15 min emerged as a good compromise where most of the signal change already materialized. The image-based IDA experiment result showed that CB7-NBD remained functional inside living cells and thus responded to the target analytes, which encouraged us to monitor the responses of more target analytes. Moreover, we aimed to explore other methods to measure changes in fluorescence signals, as the throughput of fluorescence microscopy is limited due to the necessity to analyze pixel intensity in every individual image.

Considering the successful detection of amantadine by the CB7-NBD conjugate, we were encouraged to study the behavior of the conjugate in the presence of other analytes in the cell culture medium (Fig. S4, ESI† and Table 1). For this, we first estimated the binding affinities of CB7-NBD with different analytes through fluorescent titration in a cell culture medium (Fig. S5–S13, ESI†). The estimated binding affinities are reported in Table 1. Next, we used FACS to perform IDAs with amantadine (log *K*_a_ > 8), nandrolone (log *K*_a_ = 4.03), and Phe-Gly (log *K*_a_ = 3.82) in living cells. The cells without CB7-NBD incubation were set as the negative control, and the cells treated with CB7-NBD were set as the positive control. Our results showed that the highest fluorescence quenching was achieved by amantadine, followed by nandrolone and Phe-Gly (Fig. 3C and D), which corresponded to the log *K*_a_ value trend (amantadine > nandrolone > Phe-Gly) (Table 1). In other words, the higher the log *K*_a_ value of the evaluated analyte, the stronger the fluorescence quenching effect was observed due to the competitive displacement of NBD from the cavity of the CB7 host.

**Table 1:** Binding affinities of CB7 with various analytes from fluorescence titration experiments in DMEM cell culture medium. The estimated error in log *K*_a_ is ± 0.2

Analyte	Log *K*_a_ (M^−1^)
1-Adamantanol	>8^a^
4-Fluorophenylethylamine·HCl	4.13
Amantadine·HCl	>8
C_8_mimBr_2_	5.50
Cadaverine·2HCl	3.59
Insulin	N/A
2,7-Dimethyldiazapyrenium diiodide (MDAP)	4.87
Nandrolone	4.03
Phe-Gly	3.82
Spermine·4HCl	3.35
Tyramine·HCl	3.50
Vecuronium bromide	N/A

Despite having demonstrated that FACS analysis can be used to characterize the IDAs with the CB7-NBD conjugate, its low throughput and multi-step workflow encouraged us to find a method that could allow us to test more analytes in a simpler procedure. Moreover, FACS analysis required cells to be detached from the cell culture plates with reagents such as trypsin, which can potentially interfere with IDAs by introducing a source of fluorescence quenching. In order to overcome these limitations, we tested the compatibility of CB7-NBD conjugate-based IDA with fluorescence spectrometry. Firstly, cells were treated with CB7-NBD and with the analytes (Table 1) in sequence, and then cells were washed, followed by the fluorescence intensity measurement by a microplate reader (Fig. 3E). The relative fluorescence intensity of the CB7-NBD group was set as 100%. The experiments showed that the lowest relative fluorescence intensity was achieved by amantadine and 1-adamantanol, with 9% and 14%, respectively (Fig. 3E), which are the two compounds with higher binding affinity for CB7-NBD (Table 1). The relative fluorescence intensity changes of other analytes also corresponded with the trend of their log *K*_a_ values except for MDAP (2,7-dimethyldiazapyrenium diiodide), insulin, and vecuronium bromide. This might be due to the lower cellular uptake of these three molecules since they are likely cell-internalized through other endocytosis pathways instead of passive permeation. Taken together, these results confirmed that the binding affinity-dependent competitive binding to CB7-NBD conjugate could be utilized in live cells to detect diverse target analytes. In future work, high-resolution imaging techniques such as confocal laser scanning microscopy could be used to spatially localize the CB7-NBD conjugate within the intracellular space, which could provide a more comprehensive understanding of this sensing system and broaden its scope of possible applications.

Please refer to the ESI† to get access to the materials and methods as well as the supplementary figures related to this manuscript.

## Conclusions

In this work, we investigated the potential of our previously reported unimolecular CB7-NBD conjugate to be used for the intracellular detection of molecules. The conjugate displayed exceptional cellular compatibility without detectable toxicity towards HepG2 cells and a time-dependent cellular uptake by the same cells. By performing IDAs with a variety of biomolecules of interest using a cell culture medium as the aqueous phase, we demonstrated that the CB7-NBD conjugate could respond to the target analytes in the complex live cell system, thus making it an ideal candidate for intracellular drug monitoring. Furthermore, the CB7-NBD conjugate-based sensing system is compatible with several fluorescence-measuring techniques, such as fluorescence microscopy, FACS, and fluorescence spectrophotometry with a microplate reader. This work demonstrates the great potential of CB7-dye unimolecular conjugates for label-free intracellular detection of drugs and small molecules.

## Conflicts of interest

There are no conflicts to declare.

## Supplementary Material

CB-004-D3CB00131H-s001
