# Supplementary material for: Binding affinity-based intracellular drug detection enabled by a unimolecular cucurbit[7]uril-dye conjugate
Source: RSC Chem Biol. 2023 Sep 7;4(10):760–4. doi: 10.1039/d3cb00131h (PMC10549235; doi:10.1039/d3cb00131h)
Supplement: CB-004-D3CB00131H-s001 [file CB-004-D3CB00131H-s001.pdf]

Electronic Supplementary Material (ESI) for RSC Chemical Biology.
This journal is © The Royal Society of Chemistry 2023

Supplementary Information

Binding affinity-based intracellular drug detection enabled by a unimolecular curcubit[7]uril-dye conjugate

Yanxi Liu [a,b], Changming Hu [c], Julian A. Serna [a], Frank Biedermann *[c], Pavel A. Levkin *[a,d]

[a] Karlsruhe Institute of Technology (KIT), Institute of Biological and Chemical Systems – Functional Molecular Systems (IBCS-FMS), Hermann-von-Helmholtz-Platz 1, 76344 Eggenstein-Leopoldshafen, Germany

[b] Institute of Pathology and Southwest Cancer Center, Southwest Hospital, Third Military Medical University (Army Medical University) and Key Laboratory of Tumor Immunopathology, Ministry of Education of China, 400038 Chongqing, China.

[c] Karlsruhe Institute of Technology (KIT), Institute of Nanotechnology (INT), Hermann-von-Helmholtz Platz 1, 76344 Eggenstein-Leopoldshafen, Germany

[d] Karlsruhe Institute of Technology (KIT), Institute of Organic Chemistry (IOC), Kaiserstraße 12, 76131 Karlsruhe, Germany

Corresponding author(s): frank.biedermann@kit.edu, levkin@kit.edu

Materials and Methods

Materials. All solvents were used as received from suppliers without any further purification. The concentrations of cucurbit[7]uril-tetraethylene glycol-nitrobenzoxadiazole (CB7-NBD) stock solutions were determined by fluorescence titration against a known concentration of amantadine hydrochloride by exciting the sample at 475 nm and collecting the emission intensity at 550 nm in Milli-Q water.

Fetal bovine serum (FBS), Dulbecco modified Eagle medium (DMEM), 0.25% trypsin/EDTA, 1% penicillin/streptomycin (P/S), Dulbecco's Phosphate Buffered Saline without calcium chloride and magnesium chloride (DPBS -/-) and phosphate-buffered saline (PBS) were purchased from Gibco, Life Technologies GmbH (Darmstadt, Germany). FluoroBrite™ DMEM media was obtained from Thermo Fisher Scientific (MA, USA). CellTiter 96® Non-Radioactive Cell Proliferation Assay was purchased from Promega GmbH (Walldorf, Germany).

Synthesis. The CB7-NBD conjugate was synthesized as previously described.[1] Briefly, the reporter dye NBD was attached to a TEG flexible linker, and this complex was subsequently coupled with CB7 via an Azide-Alkyne Huisgen Cycloaddition reaction. For further details regarding the synthesis, please refer to [1].

Fluorescence spectra and binding titration experiments. Steady-state emission spectra and time-resolved emission profiles were recorded on a Jasco FP-8300 fluorescence spectrometer (Jasco Deutschland GmbH, Pfungstadt, Germany) equipped with a 450 W Xenon arc lamp, double-grating excitation, and emission monochromators. Emission spectra were corrected for source intensity (lamp and grating) and the emission spectral response (detector and grating) by standard correction curves. All titration and kinetic experiments were carried out at 25 ºC using a water thermostatic cell holder STR-812, while the cuvettes were equipped with a stirrer allowing rapid mixing. For fluorescence-

based titration experiments, PMMA cuvettes with a light path of 10 mm and dimensions of 10 × 10 mm from Brand GmbH (Wertheim, Germany) with a spectroscopic cut-off at 300 nm were utilized. The binding titration isotherms were fitted with a direct binding model using our freely available thermosimfit software package (https://github.com/ASDSE). The estimated measurement and fitting errors of the reported log K_a values is ±0.2.

Cell culture. Human hepatocellular carcinoma (HepG2) cells (HB-8065™, American Type Culture Collection, VA, USA) were kindly provided by Prof. Ute Schepers (Institute of Functional Interfaces at Karlsruhe Institute of Technology, Germany). Cells were cultured in DMEM with phenol red supplemented with 10 % v/v FBS and 1 % v/v P/S (growth medium) in a humidified atmosphere at 37 °C with 5% CO_2. This same supplemented medium was used for all subsequent experiments unless otherwise stated.

Cell viability. The cell viability of CB7-NBD towards HepG2 cells was tested by a commercially-available MTT-based assay following the manufacturer's instructions (CellTiter 96®, Promega GmbH, Walldorf, Germany). HepG2 cells were seeded in 96-well plates (5 × 10^4 cells/mL, 100 µL/well) and incubated overnight to let the cells attach. Then the cells were treated with various concentrations of CB7-NBD (3.13, 6.25, 12.5, 25, 50, and 100 µM) for 72 h. After this, 10 µL of an MTT solution was added to each well and incubated at 37 °C and 5 % CO_2 for 4h. The stop solution was then added and incubated overnight at 37 °C prior to measuring absorbance at 595 nm wavelength. Cells without CB7-NBD treatment (solely cell medium) were set as the positive control, and cells exposed to cell medium with Triton X-100 at 10 % v/v were set as the negative control.

Cellular uptake. The HepG2 cells were seeded in 96-well plates (3 × 10^5 cells/mL, 100 µL/well) and incubated overnight to let the cells attach. Next, the cell medium was replaced with 100 µL fresh growth medium with CB7-NBD at a 50 µM concentration. The medium was removed at different

intervals, and the cells were washed with DPBS (-/-) thrice. The cellular uptake of CB7-NBD was then visualized with a Keyence BZ-X810 fluorescence microscope (Keyence Deutschland GmbH, Neu-Isenburg, Germany) at 20× and 60× magnifications (λ_{ex} = 470/40 nm, λ_{em} = 525/50 nm).

Analyte displacement assay. The HepG2 cells were incubated with growth medium supplemented with 50 μM CB7-NBD for 24 h. Next, the medium was removed, and the cells were successively washed three times with DPBS (-/-). Afterward, cell growth medium with 500 μM amantadine was added to each well and incubated for 15 min. Then, the green fluorescence was observed by a Keyence BZ-X810 fluorescence microscope at 20× magnification (λ_{ex} = 470/40 nm, λ_{em} = 525/50 nm) or measured by a SpectraMax iD3 microplate reader (San Jose, CA, USA).

FACS analysis. The HepG2 cells were trypsinized as cell suspension and seeded into 12-well plates at a density of 5.0×10^5 cells/well. The cells were then treated with 50 μM CB7-NBD in cell growth medium for 24 h, followed by incubation with amantadine, nandrolone, and Phe-Gly at a concentration of 500 μM in cell growth medium. Then the cells were detached, washed three times with DPBS (-/-), and immediately analyzed by FACS. The mean fluorescence intensity of 1.0×10^4 cells were analyzed for each sample.

Indicator displacement measured by a microplate reader. The HepG2 cells were trypsinized as cell suspension and seeded into 96-well plates (Corning® 96-well Half Area High Content Imaging Glass Bottom Microplate, Corning, USA) at a density of 1.5×10^4 cells/well. The cells were then cultured overnight to let the cells attach to the plate and then treated with 50 μM CB7-NBD in cell growth medium for 24 h, followed by incubation with diverse analytes (Table 1) at the concentration of 500 μM in cell growth medium. The cells were washed with DPBS (-/-) three times and immediately analyzed by a microplate reader (SpectraMax® iD3 plate reader, CA, USA) for fluorescence intensity.

A.

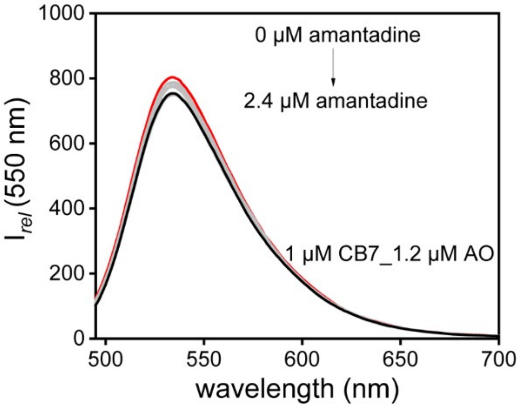

B.

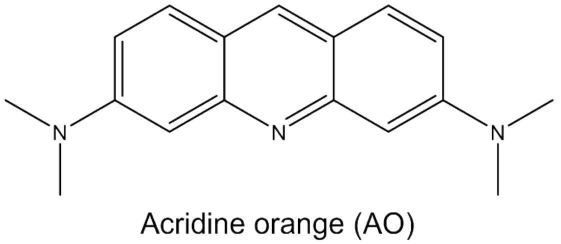

Figure S1. (A) Emission spectra of 1 μM CB7 and 1.2 μM acridine orange (AO) (λ_{ex} = 480 nm) upon addition of amantadine (0 to 2.4 μM) in DMEM cell culture media. Please be aware that cell culture media inherently exhibits chromophoric and emissive properties. As such, potential alterations to the absorbance or emission signal of the chemosensors, induced by the addition of an analyte, may be seen only as emerging or vanishing spectral shoulders. (B) Chemical structure of AO.

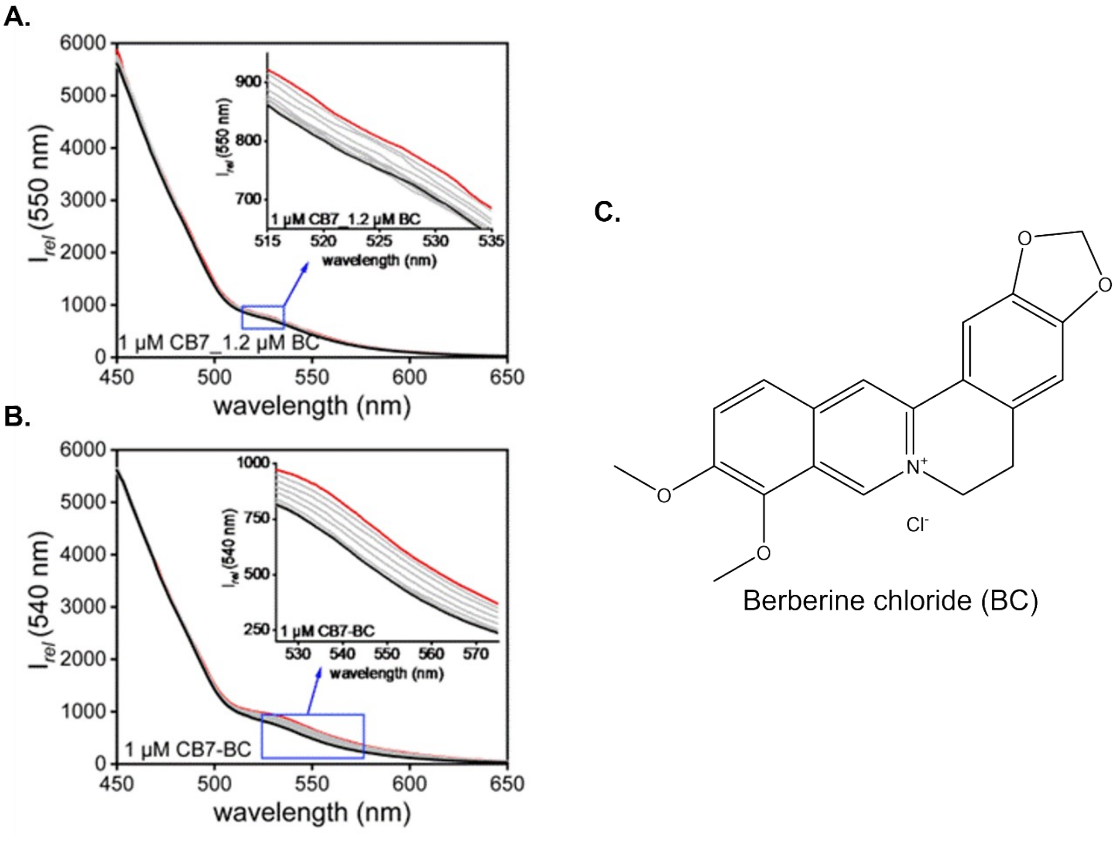

Figure S2. (A) Emission spectra of 1 µM CB7 and 1.2 µM berberine chloride (BC) (λ_{ex} = 350 nm) (non-covalent bonding) upon addition of amantadine (0 to 2.4 µM) in DMEM cell culture media. (B) Emission spectra of the covalent CB7-BC conjugate at a concentration of 1 µM (λ_{ex} = 350 nm) upon addition of amantadine (0 to 2.4 µM) in DMEM cell culture media. Please be aware that cell culture media inherently exhibits chromophoric and emissive properties. As such, potential alterations to the absorbance or emission signal of the chemosensors, induced by the addition of an analyte, may be seen only as emerging or vanishing spectral shoulders. (C) Chemical structure of BC.

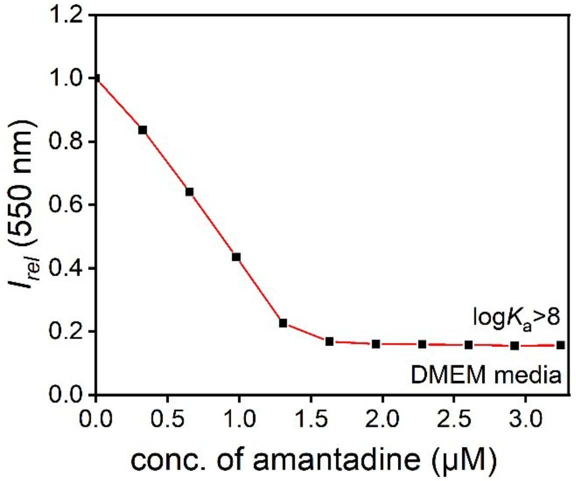

Figure S3. CB7-NBD ⊃ amantadine. Fitting plot of normalized emission intensity at 550 nm of 1 µM CB7-NBD in DMEM media at 25 ºC, upon addition of amantadine, λ_{ex} = 475 nm. Intervals between titration steps: 50 seconds.

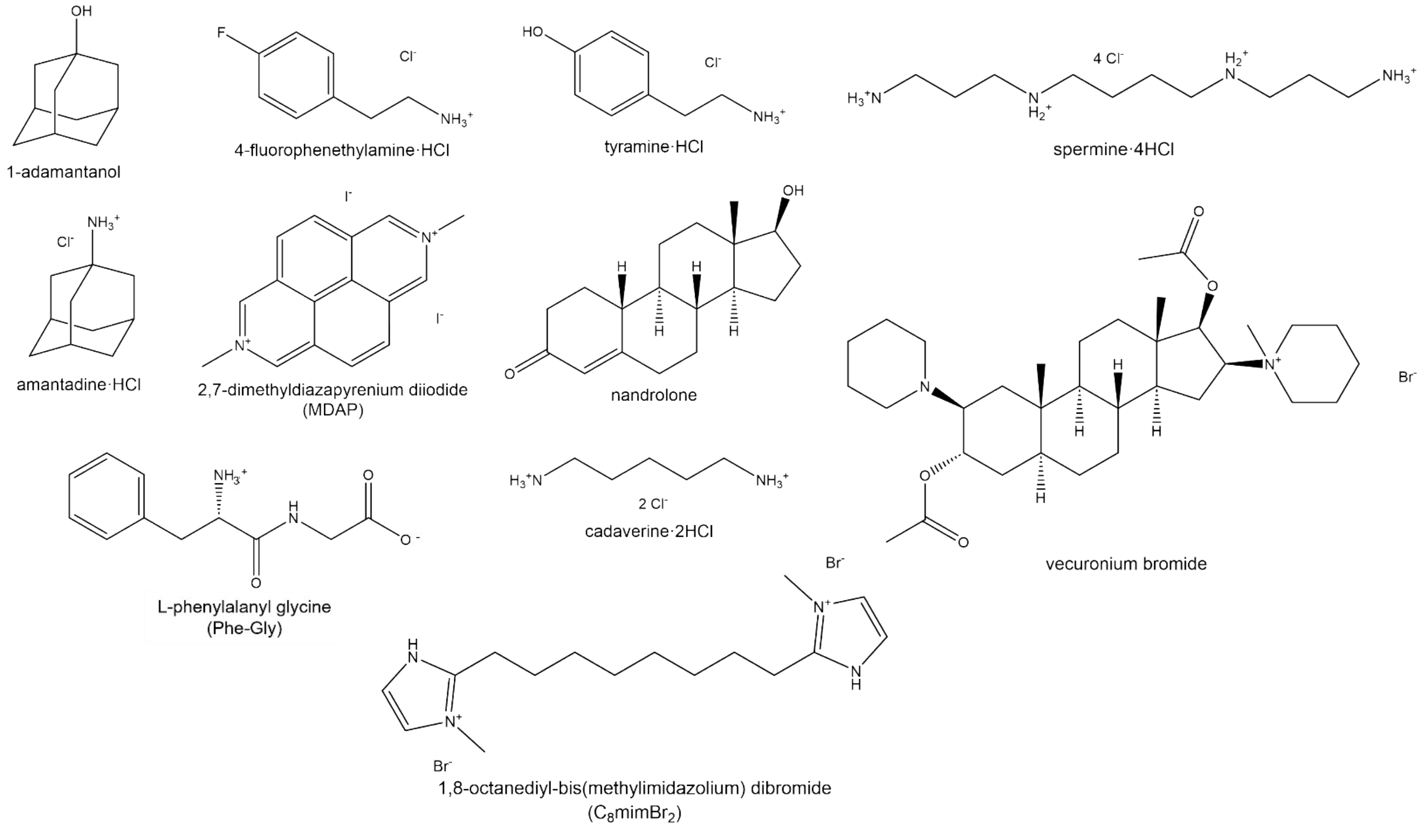

Figure S4. Chemical structures of the compounds used as analytes for the IDAs with the CB7-NBD unimolecular conjugate.

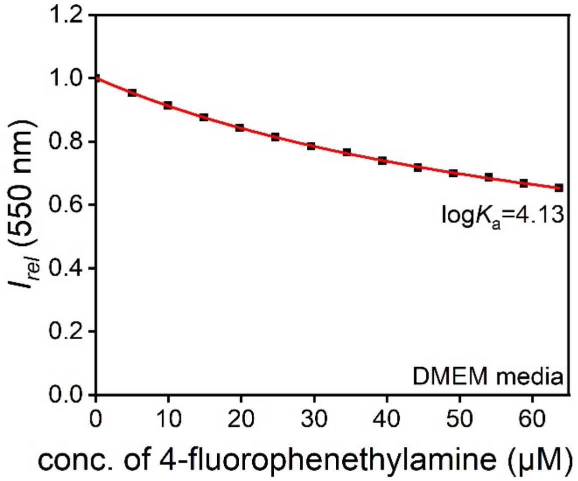

Figure S5. CB7-NBD ⊃ 4-fluorophenethylamine. Fitting plot of normalized emission intensity at 550 nm of 1 μM CB7-NBD in DMEM media at 25 °C, upon addition of 4-fluorophenethylamine, λ_{ex} = 475 nm. Intervals between titration steps: 500 seconds.

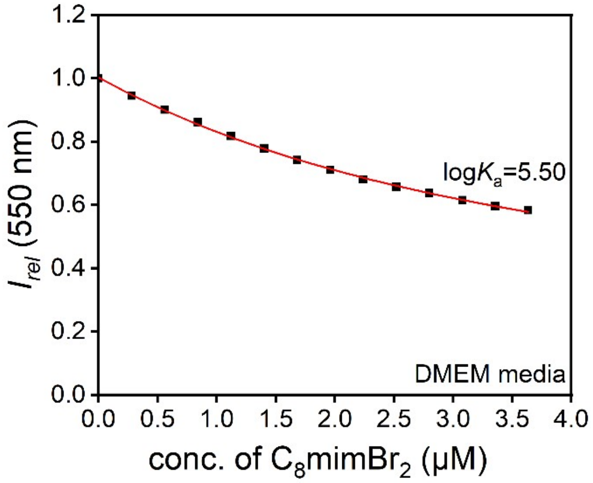

Figure S6. CB7-NBD ⊃ C_8mimBr_2. Fitting plot of normalized emission intensity at 550 nm of 1 μM CB7-NBD in DMEM media at 25 °C, upon addition of C_8mimBr_2, λ_{ex} = 475 nm. Intervals between titration steps: 50 seconds.

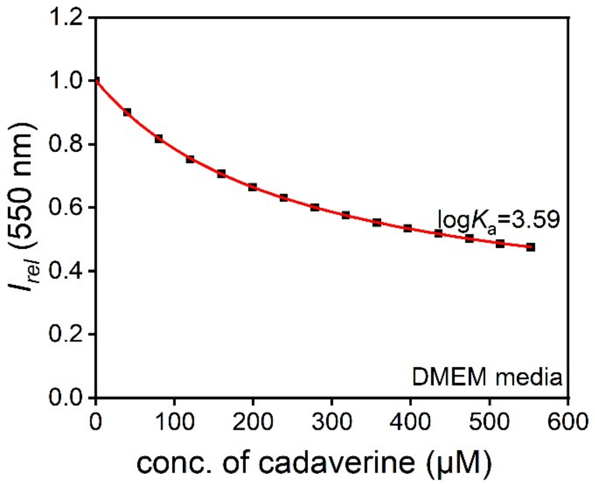

Figure S7. CB7-NBD ⊃ cadaverine·HCl. Fitting plot of normalized emission intensity at 550 nm of 1 μM CB7-NBD in DMEM media at 25 ºC, upon addition of cadaverine·HCl, λ_{ex} = 475 nm. Intervals between titration steps: 50 seconds.

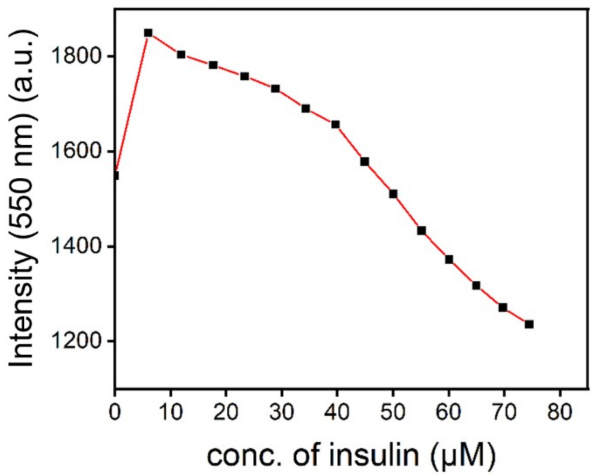

Figure S8. CB7-NBD ⊃ insulin. Plot of emission intensity at 550 nm of 1 μM CB7-NBD in DMEM media at 25 ºC, upon addition of insulin, λ_{ex} = 475 nm. Intervals between titration steps: 250 seconds. The lines connect the data points to guide the eye and do not represent a fitting curve.

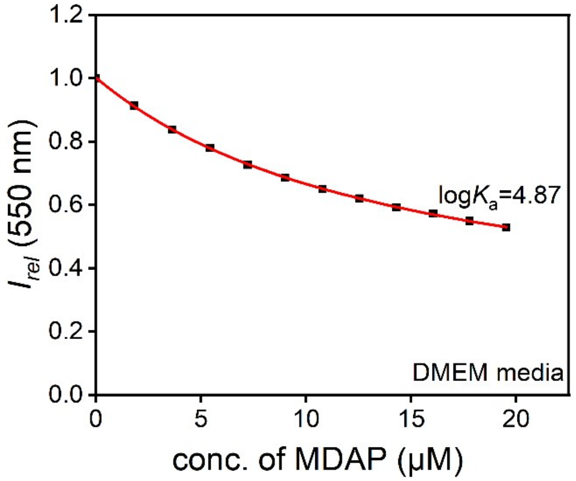

Figure S9. CB7-NBD ⊃ MDAP. Fitting plot of normalized emission intensity at 550 nm of 1 μM CB7-NBD in DMEM media at 25 ºC, upon addition of MDAP, λ_{ex} = 475 nm. Intervals between titration steps: 50 seconds.

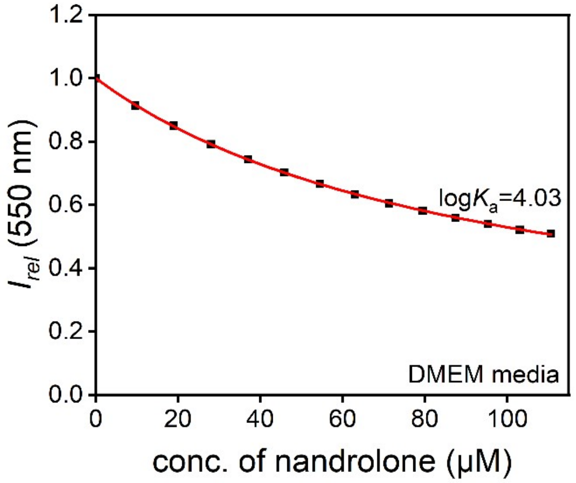

Figure S10. CB7-NBD ⊃ nandrolone. Fitting plot of normalized emission intensity at 550 nm of 1 μM CB7-NBD in DMEM media at 25 ºC, upon addition of nandrolone, λ_{ex} = 475 nm. Intervals between titration steps: 50 seconds.

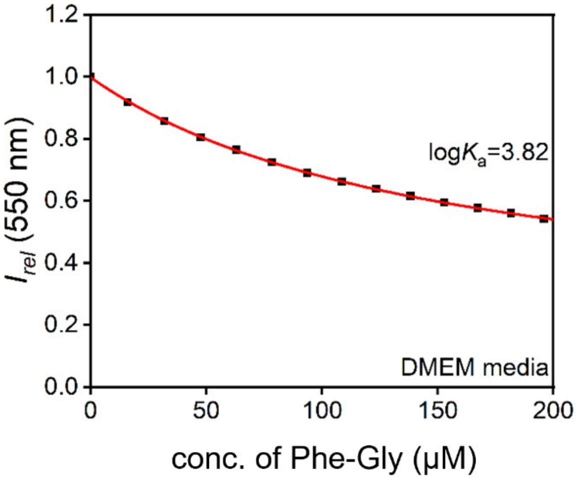

Figure S11. CB7-NBD ⊃ Phe-Gly. Fitting plot of normalized emission intensity at 550 nm of 1 μM CB7-NBD in DMEM media at 25 ºC, upon addition of Phe-Gly, λ_{ex} = 475 nm. Intervals between titration steps: 50 seconds.

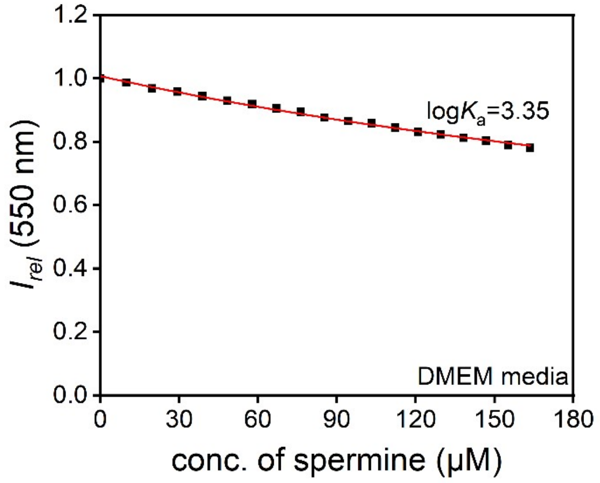

Figure S12. CB7-NBD ⊃ spermine. Fitting plot of normalized emission intensity at 550 nm of 1 μM CB7-NBD in DMEM media at 25 ºC, upon addition of spermine, λ_{ex} = 475 nm. Intervals between titration steps: 250 seconds.

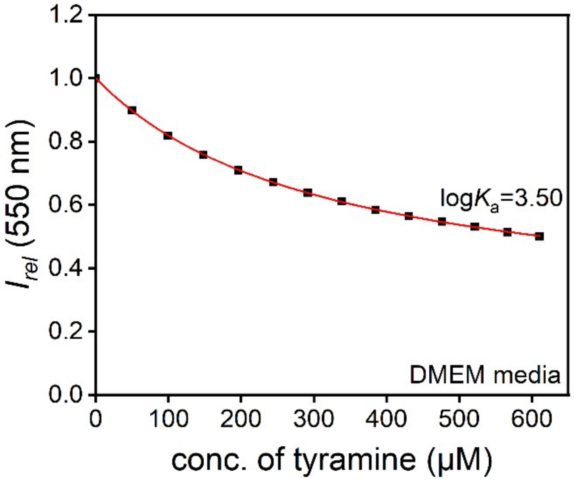

Figure S13. CB7-NBD ⊃ tyramine. Fitting plot of normalized emission intensity at 550 nm of 1 μM CB7-NBD in DMEM media at 37 ºC, upon addition of tyramine, λ_{ex} = 475 nm. Intervals between titration steps: 500 seconds.

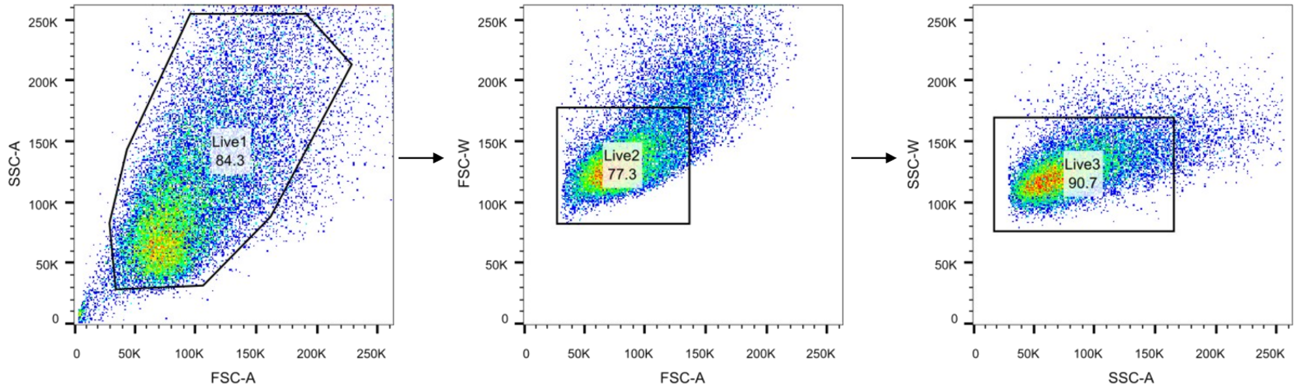

Figure S14. FACS scatter plots of HepG2 cells incubated with CB7-NBD after exposure to sole culture medium (control). The refinement of the cellular population of interest was performed by defining multiple gates or regions of interest (ROI) according to their forward and side scatter area and width parameters. The green fluorescence histogram of the control (Figure 3C, main manuscript) was calculated from the ROI depicted in the scatter plot on the right.

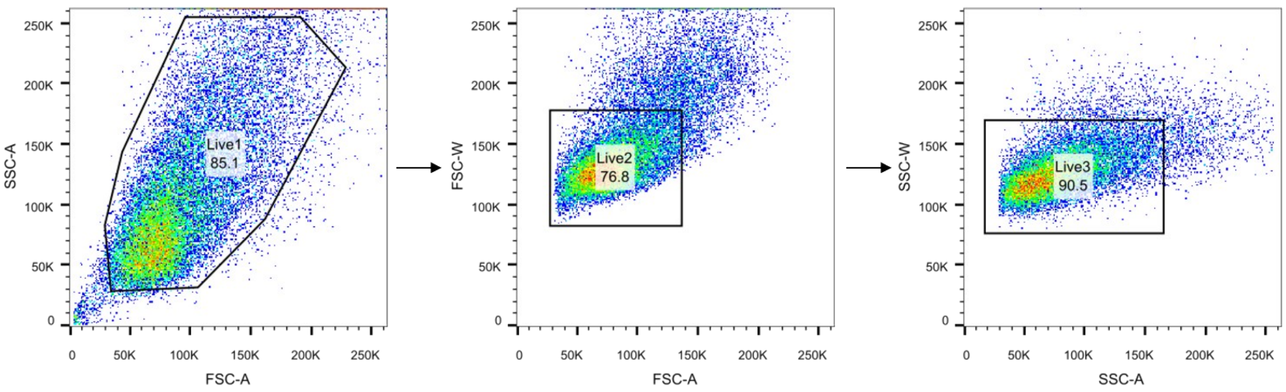

Figure S15. FACS scatter plots of HepG2 cells incubated with CB7-NBD after exposure to amantadine in cell culture medium. The refinement of the cellular population of interest was performed by defining multiple gates or regions of interest (ROI) according to their forward and side scatter area and width parameters. The green fluorescence histogram of the amantadine group (Figure 3C, main manuscript) was calculated from the ROI depicted in the scatter plot on the right.

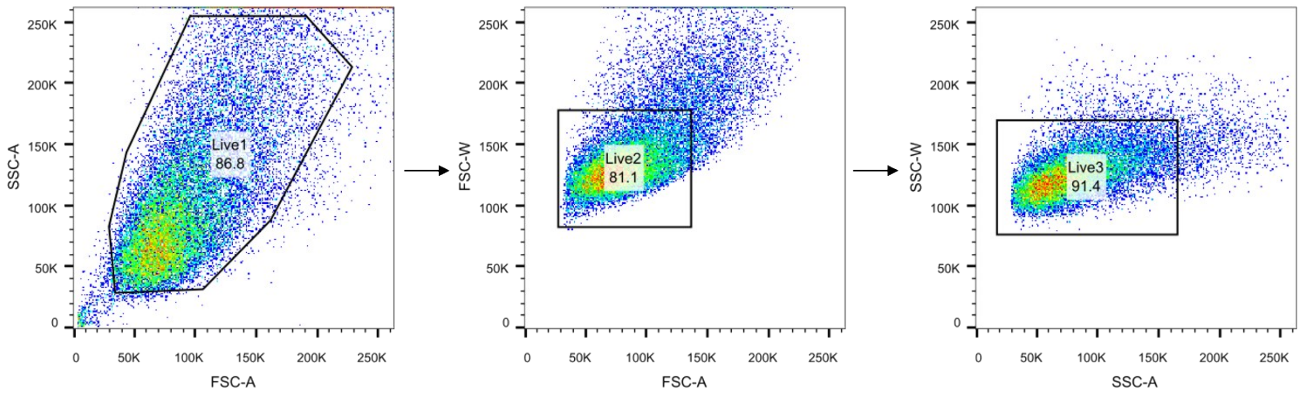

Figure S16. FACS scatter plots of HepG2 cells incubated with CB7-NBD after exposure to nandrolone in cell culture medium. The refinement of the cellular population of interest was performed by defining multiple gates or regions of interest (ROI) according to their forward and side scatter area and width parameters. The green fluorescence histogram of the nandrolone group (Figure 3C, main manuscript) was calculated from the ROI depicted in the scatter plot on the right.

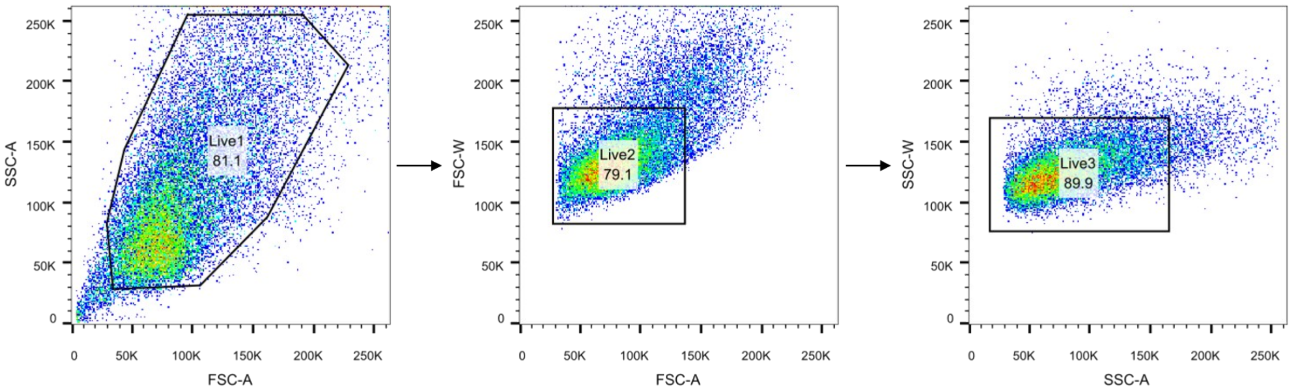

Figure S17. FACS scatter plots of HepG2 cells incubated with CB7-NBD after exposure to Phe-Gly in cell culture medium. The refinement of the cellular population of interest was performed by defining multiple gates or regions of interest (ROI) according to their forward and side scatter area and width parameters. The green fluorescence histogram of the Phe-Gly group (Figure 3C, main manuscript) was calculated from the ROI depicted in the scatter plot on the right.

References

1 C. Hu, T. Jochmann, P. Chakraborty, M. Neumaier, P. A. Levkin, M. M. Kappes and F. Biedermann, *J. Am. Chem. Soc.*, 2022, **144**, 13084–13095.
